# Integrative genomic and transcriptomic approaches decipher pre-harvest sprouting resistance in rice

**DOI:** 10.3389/fpls.2025.1692942

**Published:** 2025-12-11

**Authors:** Lv Yang, Renyuan Yang, Yuxuan Wang, Muhammad Asad Ullah Asad, Yueying Wang, Zhiyuan Chang, Tianhui Miao, Shudong Yang, Yiting Wei, Shanshan Wu, Jiaxue Bao, Mingming Wu, Jing Ye, Rongrong Zhai, Shenghai Ye, Xiaoming Zhang, Faliang Zeng, Faming Yu

**Affiliations:** 1 Institute of Crop and Nuclear Technology Utilization, Zhejiang Academy of Agricultural Science, Hangzhou, China; 2 Wenzhou Vocational College of Science and Technology, Wenzhou, China; 3 State Key Laboratory of Rice Biology, China National Rice Research Institute, Hangzhou, China; 4 Shengzhou Seed Multiplication Farm, Shengzhou, China; 5 Longyou Agriculture and Rural Affairs Bureau, Quzhou, China; 6 Jiangxi Academy of Agricultural Sciences Rice Research Institute, Nanchang, China

**Keywords:** rice, pre-harvest sprouting, GWAS, transcriptome analysis, molecular breeding

## Abstract

Pre-harvest sprouting (PHS) seriously compromises rice yield and quality, increase susceptibility to insect pest and reduce seed viability. Beside agronomic control measures, the genetic makeup of rice plants serves as a fundamental determinant in conferring resistance to PHS. Therefore, integrating multi-omics strategies to construct high-resolution genetic variation maps, screen extreme-phenotype germplasm, and identify causal genes are pivotal for generating PHS-resistant breeding material. In this study, we performed whole-genome re-sequencing of 165 highly diverse indica rice accessions to construct a high-density genetic variation map, obtaining a dataset comprising 1,584,905 high-quality SNPs for subsequent association analysis. Genome-wide association studies (GWAS) further uncovered 21 candidate loci and multiple candidate genes associated with PHS, from which key candidate genes were prioritized. In particular, previously cloned PHS-related genes—*OsCDP3.10*, *OsWRKY50*, *UGT74J1*, *OsJAZ6*, and *IPA1*. Additionally, we investigated the transcriptional analyses in cultivars Z33 and Z216 under high-humidity conditions and identified 19,087 differentially expressed genes (DEGs). Notably, by integrating GWAS and transcriptomic analyses, we identified *UGT74J1* as a promising candidate gene, and haplotype analysis further revealed *UGT74J1*-Hap3 as a superior haplotype associated with PHS resistance. This multi-omics dataset and the candidate genes identified will provide valuable genetic resources for molecular breeding toward improved PHS resistance in rice.

## Introduction

Pre-harvest sprouting (PHS) is a phenomenon where mature grain crops may sprout prematurely in their panicles if not harvested in time, especially under high-temperature and high-humidity conditions (Chen et al., 2021). With global climate change, the frequency of extreme weather events has increased, leading to more unpredictable rainfall and high-humidity conditions during harvest (Tai et al., 2021). Consequently, the incidence of PHS in rice (*Oryza sativa* L.). has significantly increased. This phenomenon directly diminishes grain yield and quality, leading to substantial economic losses and jeopardizing food security, especially in humid rice-growing areas.

Seed dormancy is a key agronomic trait governing PHS resistance in rice. Varieties with strong dormancy can maintain a quiescent state for an extended period after seed maturation, which prevents germination under permissive conditions and thereby confers enhanced PHS resistance. A central challenge in contemporary rice breeding lies in orchestrating a precise equilibrium between robust seed dormancy and rapid, uniform germination (Holdsworth et al., 2008). Consequently, the strategic development of novel cultivars endowed with an optimal dormancy profile is imperative for the dual objectives of sustaining yield potential and preserving grain quality. This complex agronomic trait is governed by a sophisticated molecular regulatory network (Finkelstein et al., 2008; Gubler et al., 2005). The regulatory mechanisms governing seed dormancy and seed vigor critically influence crop yield and grain quality through complex interactions involving phytohormonal signaling, environmental stimuli, and seed physiological status. Several phytohormones such as Abscisic acid (ABA), jasmonic acid (JA), and brassinosteroids (BR) serve as pivotal regulators orchestrating dormancy establishment and sustainability (Khan, 2025; Thilakarathne et al., 2025). These hormones coordinate intra-seed signaling networks to precisely modulate dormancy depth and germination competency (Wang et al., 2020a). ABA suppresses precocious germination under adverse conditions by inducing stomatal closure to minimize water dissipation, thereby maintaining seed desiccation status (Chen et al., 2022; Fang et al., 2008). JA potentiates dormancy intensity, enabling seeds to retain quiescence even in permissive environments, consequently enhancing seed viability and ecological adaptability (Dave et al., 2011; Liu et al., 2019). BR dynamically regulate the dormancy-germination transition through modulation of endogenous hormonal homeostasis and acceleration of water uptake dynamics (Xiong et al., 2022).

The molecular architecture governing pre-harvest sprouting constitutes an intricate regulatory network entailing synergistic interactions among multiple genetic components (Wang et al., 2020b). *OsMFT2* (Mother of FT and TFL2) belongs to phosphatidylethanolamine-binding protein (PEBP) family and regulate flowering time and seed dormancy. *OsMFT2* exhibits seed-specific expression in rice and enhances ABA signaling through interacting with downstream proteins and regulating expression and activity of transcription factors (TFs) *OsbZIP23*, *OsbZIP66*, and *OsbZIP72*, thereby regulating seed dormancy and germination processes (Song et al., 2020). The C2H2-type zinc finger protein encoded by *OsZFP15* accelerates seed germination via regulating the increase of ABA catabolism (Wang et al., 2022). Under ABA accumulation, *SAPK10* phosphorylates TF *bZIP72* at Ser71, which both stabilizes the *bZIP72* protein and augments its binding affinity for the promoter of AOC, —a key JA biosynthesis gene (Kobayashi et al., 2005, Kobayashi et al., 2004). This regulatory cascade consequently elevates endogenous JA levels and suppresses germination (Wang et al., 2022). Moreover, loss activity of *OsBZR1*, a core component of BR signaling, *OsBZR1* due to functional mutations, moderately attenuate coleoptile elongation but significantly enhance pre-harvest sprouting resistance through delayed germination (Xiong et al., 2022).

Identification of PHS-resistant genes, superior haplotypes, and associated markers are the crucial objectives in rice breeding to enhance crop resilience and reduce grain yield losses (Aloryi et al., 2025). High-throughput functional marker-assisted selection enables the precise identification of resistant germplasm and significantly boost breeding efficiency (Robinson et al., 2010). This approach accelerates the introgression of favorable alleles, enabling the rapid development of broadly adaptable, high-yielding rice varieties with enhanced stability against environmental adversities (Yi et al., 2025). The development of such varieties is vital for ensuring global food security in the face of increasing population (Zhang et al., 2020; Zhu et al., 2019). Meanwhile, multidimensional regulatory networks of PHS obtained by integrating genomic, transcriptomic, and population analyses, not only advances gene discovery and functional validation but also enhances our mechanistic understanding of PHS (Min et al., 2024). Consequently, this PHS regulatory network provides a robust theoretical foundation for genomic selection and intelligent design in rice breeding.

The integration of multi-omics analyses utilizing high-density SNPs in plant populations has emerged as a robust strategy for identifying candidate genes (Lv et al., 2022). The advent of methodologies such as GWAS, transcriptomics, metabolomics, and proteomics has provided powerful tools for systematically elucidating the molecular mechanisms underlying PHS resistance. Compared to traditional linkage analysis, GWAS can detect a broader spectrum of alleles and genetic variations (Min et al., 2021). For instance, the combined use of GWAS and linkage mapping identified *Sdr4* as a key regulator of PHS in rice. This gene exhibits additive effects with *Rc* and *SD1*, its genotypic distribution shows significant association with climatic conditions, and molecular markers have been developed for breeding applications (Zhao et al., 2022). Furthermore, integrated transcriptomic and metabolomic analyses revealed that the *SbPP2C33* gene influences seed germination in sorghum by modulating ABA signaling and carbohydrate mobilization, providing new insights into the molecular regulatory network of PHS (Ju et al., 2025). In this study, whole-genome resequencing of 165 diverse indica rice accessions was conducted to generate a high-density genetic variation map from high-quality SNPs. By integrating GWAS and transcriptome profiling, we identified multiple significant PHS-associated loci and candidate QTLs. Further multi-omics integration revealed critical PHS-related regulatory pathways and differentially expressed genes. Our finding establishes a comprehensive molecular framework for understanding PHS and provide valuable genetic targets and technical foundations for breeding PHS-resistant rice varieties.

## Materials and methods

### Plant materials

The indica population was selected for its high genetic diversity and direct agricultural relevance. An assemblage of 165 *indica* accessions was established for this study (**Supplementary Table 1**). These genetic resources are maintained at the Zhejiang Academy of Agricultural Sciences (ZAAS, Hangzhou, Zhejiang Province, China) and cultivated in ZAAS experimental fields.

### Statistical analysis of phenotypic variation

A panel of 165 *indica* rice varieties was assessed for panicle sprouting evaluation in 2024. Main panicles were collected from mature plants at harvest, 35 days after heading. For each variety, main panicles from three plants were randomly sampled as biological replicates. The collected panicles were soaked in water for 3 hours, then drained, gently dried, and transferred to the artificial climate chamber at ZAAS under a 12/12-hour light/dark cycle with temperatures of 30 °C and 25 °C, respectively. The relative humidity was set to > 90% for the high-humidity treatment group and 70% for the control group (Liu et al., 2022; Zhao et al., 2022). Germination rates, indicated by the emergence of white sprouts, were recorded at 24-hours interval. The final result for each accession was calculated as the average across the three replicates. The descriptive statistical analysis was performed in R 4.0.3 software. Based on the phenotypic data, the traits were validated for the analysis by GraphPad Prism.9 software. The significant differences between the control and treatment of the samples were analyzed by Student’s *t* test. The results with *P* < 0.05 and *P* < 0.01 were considered statistically significant and denoted by one star and two stars, respectively.

### Variant calling

For each accession, DNA sequencing was carried out on a single individual. Total DNA was extracted from the leaves of one-month-old rice plants and subjected to genome sequencing with 15× coverage on the DNBSEQ-T7 platform. Fastp (v0.23.2) with default parameters was used for sequencing data quality control (Chen, 2023). The processed reads were aligned to the Nipponbare reference genome (MSU v7.0) using bwa (v0.7.17-r1188) (mem-M-R-K 10000000). samtools (v1.9) facilitated format conversion and index construction (Li et al., 2009). Variant calling employed GATK4 (HaplotypeCaller-ERC GVCF), with CombineGVCFs for merging gvcf files and GenotypeGVCFs for genotyping (Heldenbrand et al., 2019). Quality control of single nucleotide polymorphisms (SNPs) utilized GATK4’s variant filter (QD2, QUAL30, FS60, MQ40, MQRankSum-12.5, ReadPosRankSum-8). SNP filtering was accomplished by vcftools (maf 0.05, max missing 0.8) (Danecek et al., 2011).

### Population structure analysis and LD analysis

A total of 1,584,905 high-quality SNPs were filtered and used for population structure analysis. SnpEff (V4.3m) was employed for functional annotation of the population genotypes (Kawahara et al., 2013). Based on SNP site information, population principal component analysis (PCA) was performed using plink (v1.90b6.21), population structure was assessed using mixture v1.3.0 (Alexander and Lange, 2011), and a phylogenetic tree was constructed using iqtree v2.2.5 (Minh et al., 2020). Visualization was carried out using iTol (http://itol.embl.de/).

Linkage disequilibrium (LD) decay analysis was performed for the entire population using PopLDecay with the following parameters: -MaxDist 500, -MAF 0.05, -Het 0.88, and -Miss 0.999 (Zhang et al., 2019).

### Genome-wide association study

The PHS traits were measured. For genome-wide association analysis (GWAS) of 165 *indica* accessions, we used the Farm CPU, MLM, and MLMM (PC = 5, vc.method=“BRENT”, maxLoop=20, method.bin=“static”, priority=“speed”) models from the R package rMVP (Kang et al., 2010). To correct for population structure, PCA was first conducted using PLINK, and the top five PCs were incorporated as covariates. Manhattan and Quantile-Quantile (Q-Q) plots illustrating the GWAS results were generated using the R package ‘qqman’ (v1.4.5) in R version 3.4.2. A total of 1,584,905 high-quality SNPs with missing data ≤20% and MAF ≥0.05 were selected (Visscher et al., 2012). The significant association threshold was -lg(*p*) =5 (Gao et al., 2020; Lv et al., 2022; Wei et al., 2021). Based on the linkage disequilibrium (LD) decay threshold (r^2^ = 0.2, Distance =250Kb), candidate genes were defined as those flanking 200 kb upstream and downstream of the significant SNPs (Kang et al., 2010; Klinsawang et al., 2024; Lv et al., 2021).

### Transcriptome analysis

For transcriptomic analysis, seeds from cultivars Z216 and Z33 were collected from spikes at 24 hours post-germination. As described in the phenotypic investigation, samples from three experimental replicates were pooled to form three biological replicates per cultivar. Total RNA was extracted from these pooled samples using TRIzol reagent. Based on RNA integrity (RIN >6) and purity (concentration >50 ng/μL), per sample were selected for subsequent RNA-seq library construction and sequencing (Lv et al., 2022; Wei et al., 2024). Raw data were quality-controlled with fastp (v0.23.2). The processed reads were aligned to the Nipponbare reference genome (Msu7.0) using HISAT2 (v2.2.1) (Kim et al., 2015) with parameters (-dta -t -p 1) and indexed with samtools (v1.9) (Li et al., 2009). Gene expression was normalized using FPKM (Trapnell et al., 2012). featureCounts (v2.0.1) quantified reads with parameters (-T 2 -p -t exon -g gene_id) (Liao et al., 2014).

Differentially expressed genes (DEGs) between the control and treatment groups of cultivars Z216 and Z33 were identified using the edgeR package (Robinson et al., 2010). DEGs were identified based on stringent thresholds (CPM >1, bcv = 0.2, *P*-value <0.01, |log_2_FC| ≥2) (Robinson et al., 2010). Genes consistently differentially expressed in both replicates were considered core DEGs and annotated using TBtools through GO and KEGG analysis (Chen et al., 2020).

### Haplotype analysis

Candidate genes were annotated with snpEff (v4.3m) based on the variation map of 165 samples. Variants annotated as missense, frameshift, and stop-gain mutations were selected, followed by further screening for genotype differences within the coding regions of the corresponding genes. Haplotypes were then correlated with phenotypes to identify superior haplotypes (Hothorn et al., 2008). The significant differences between different haplotypes were analyzed by Student’s *t* test. The results with *P* < 0.05 and *P* < 0.01 were considered statistically significant and denoted by one star and two stars, respectively (Wei et al., 2024; Lv et al., 2022).

## Results

### Evaluation of PHS phenotypes

**Figure 1:**
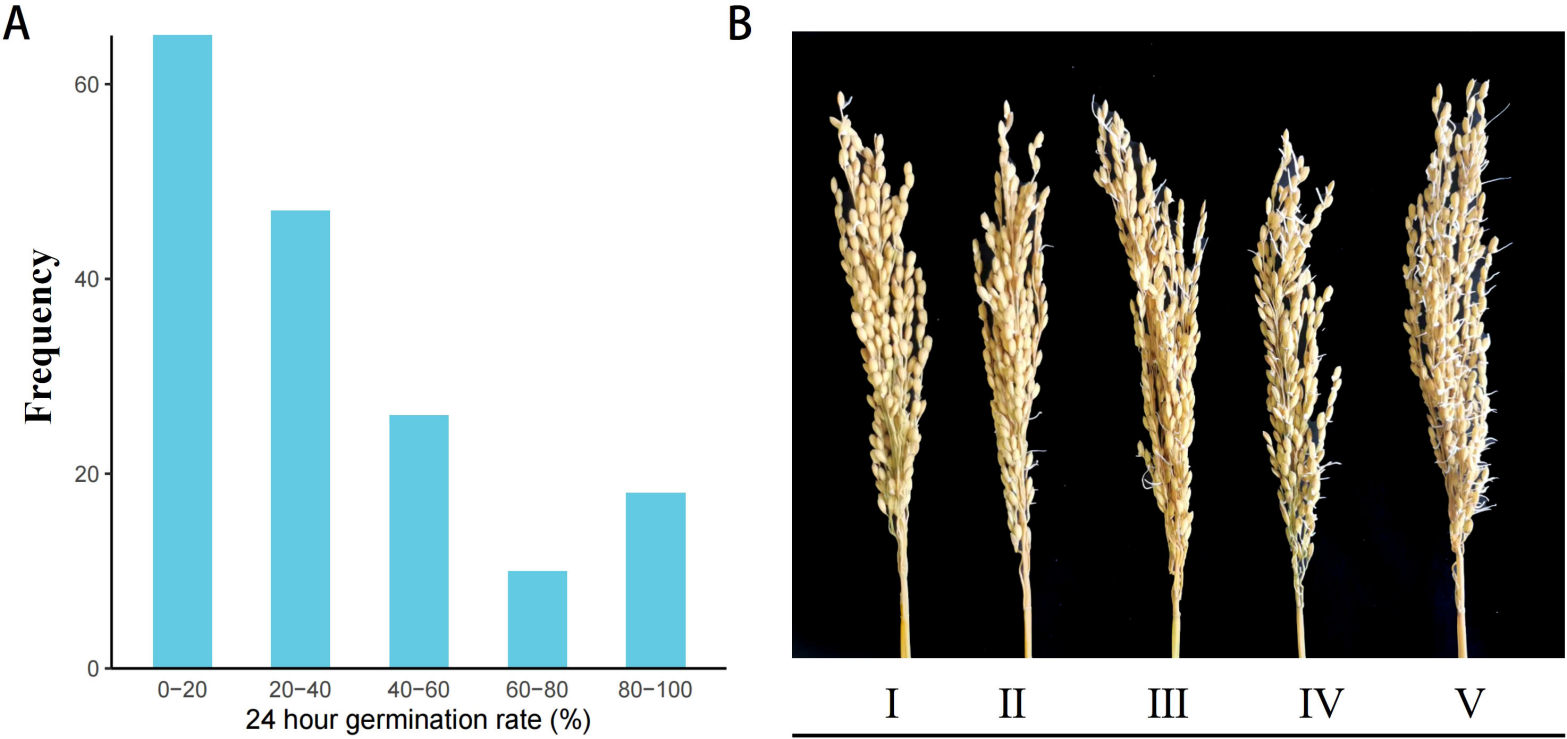
Comparative phenotypic difference of PHS in 165 *indica* accessions. **(A)** Frequency distribution of 24-hour PHS rate in the population; **(B)** Phenotypic characteristics of varieties with different PHS types. Varieties were classified into PHS types I-V based on the spike germination rate, with each grade representing an approximately 20% increment.

We evaluated the pre-harvest sprouting (PHS) phenotypes in 165 *indica* accessions to identify germplasms with extreme PHS traits and to uncover novel candidate genes with PHS resistance. Seeds were exposed to a controlled artificial climate chamber simulating a high-humidity maturation environment. The dynamic progression of pre-harvest sprouting (PHS) was monitored at 24-hour intervals. Based on the calculated panicle germination rate, cultivars were categorized by PHS severity. This classification revealed 18 susceptible cultivars (80–100% germination), such as Ai Bai Qiu and Zhe Zao 33, and 64 resistant cultivars (0–20% germination), including Guang Xuan 3 Hao (**Figure 1A**). The results indicated significant variation in PHS susceptibility among the accessions (**Figure 1B**).

### Genomic variation and population structure analysis

**Figure 2:**
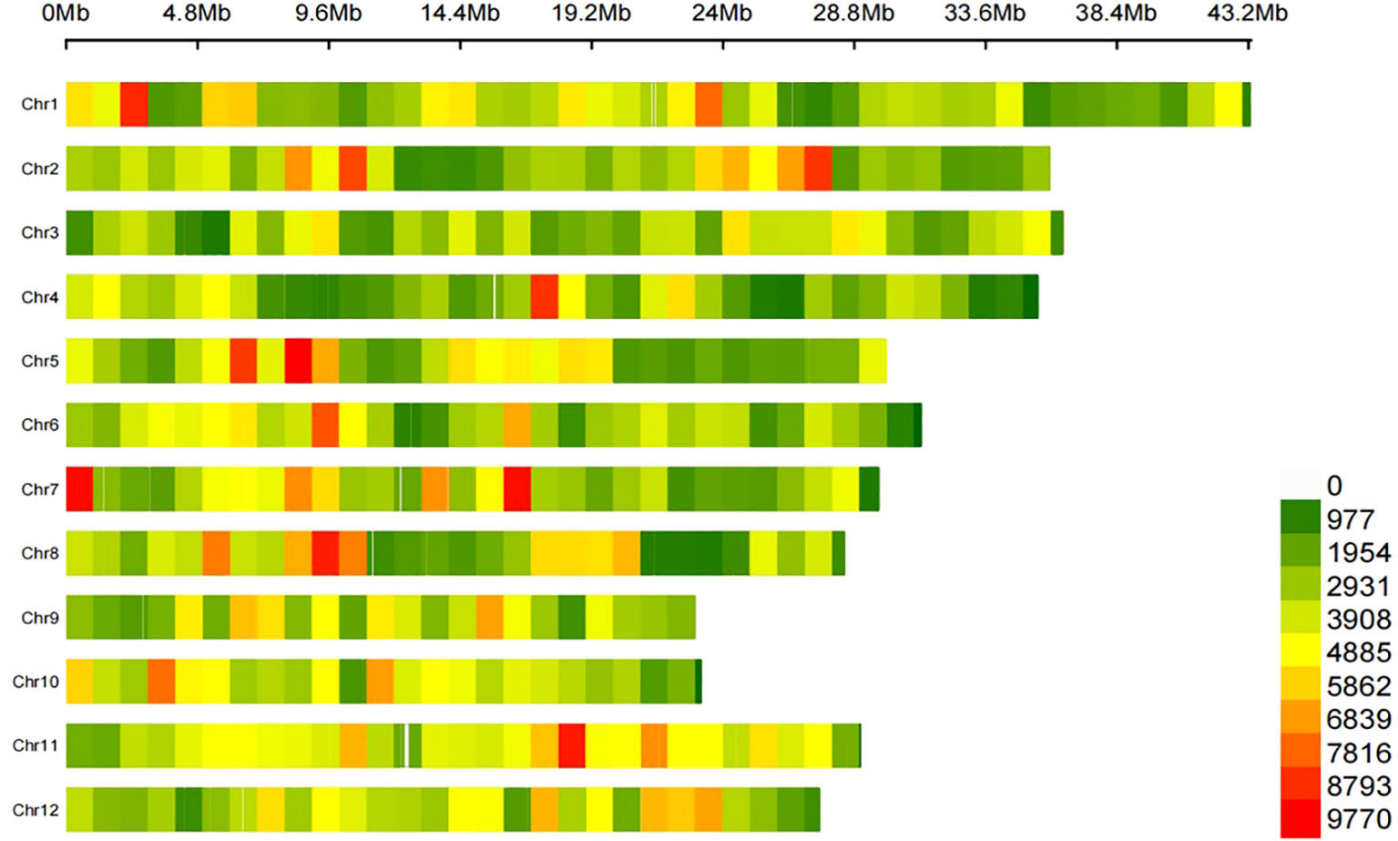
Identification of genomic variations in 165 *indica* accessions. The legend indicates the density of SNPs.

To identify the variants and candidate genes associated with PHS, we re-sequenced 165 *indica* accessions, generating 799 Gb total sequencing length with an average depth of ~13×. After evaluating/filtering, we obtained 1,584,905 high-quality SNPs covering the whole genome, with 4.25 SNPs per kb on average (**Figure 2**). SNP distribution showed that 22.32% were in intergenic regions, 63.11% within 2 kb of transcription start/end sites, and 14.56% in gene bodies. Chromosome 1 had the most SNPs (176,605 sites), while chromosome 9 had the fewest (97,806 sites). These sites provide valuable data sets that significantly advances rice biology and breeding research.

**Figure 3:**
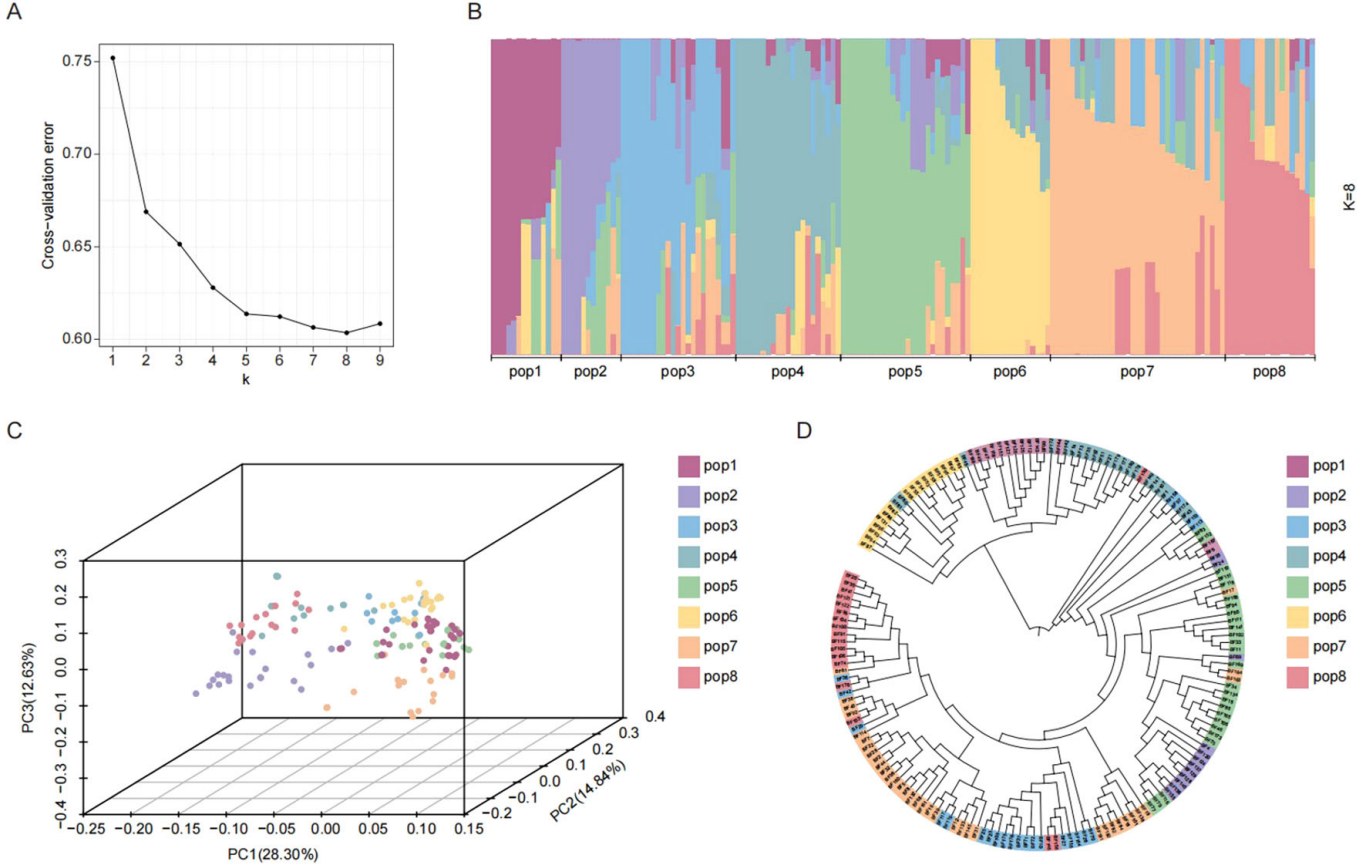
Population structures analysis. **(A)** Cross validation error of population structure; **(B)** Population structure analysis; **(C)** Principal component analysis; **(D)** Construction of phylogenetic trees. Eight rice subpopulations indicated with different colors.

Using filtered SNPs, we analyzed the population structure of 165 *indica* accessions to assess genetic diversity and relationships. Optimal population structure analysis (K = 8) classified the 165 *indica* accessions into eight distinct subpopulations (pop1–pop8) (**Figures 3A, B**). Given the minor structural differences within the population, we further assessed it using SNP-based principal component analysis (PCA). The results showed that the first 3 principal components among the top 10 only explained 55.77% of the variation, while principal components 4 to 10 had similar variation explanation rates. This indicates that there are no single or few dominant genetic variation dimensions that can strongly differentiate the subpopulations within this group (**Figure 3C**). Phylogenetic reconstruction using the NJ method also resolved eight primary clusters (**Figure 3D**). This observed multi-dimensionality underscores the complex and diverse genetic architecture of the rice population.

### GWAS

**Figure 4:**
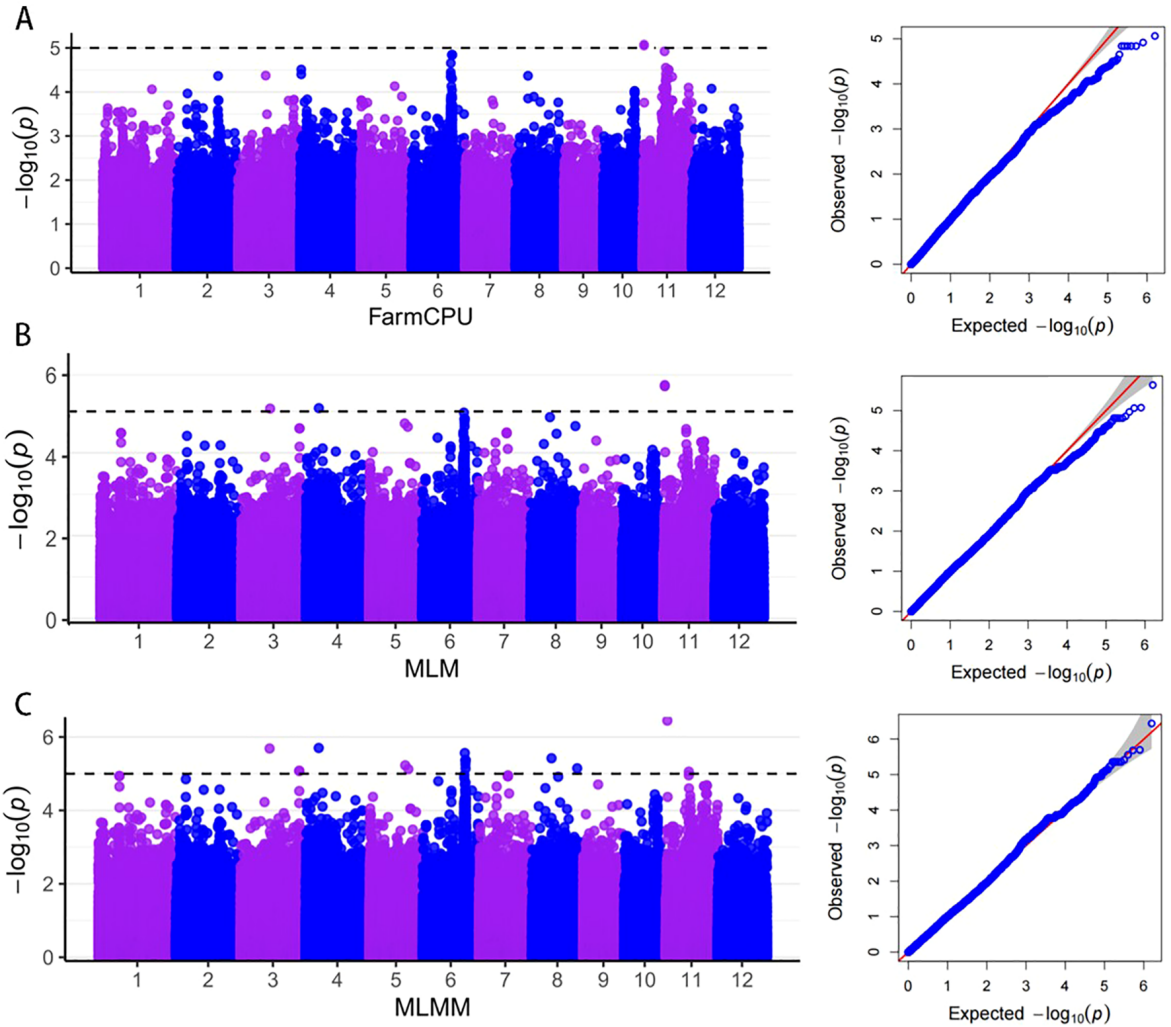
Genome-wide association analysis based on PHS rates. **(A–C)** are Manhattan and QQ plots of GWAS FarmCPU, MLM, and MLMM models.

A total of 21 significant loci were identified through GWAS for PHS (**Figure 4**). The MLMM, MLM, and FarmCPU models contributed 17, 3, and 1 locus, respectively. Importantly, three loci were co-detected by at least two models, highlighting their consistent genetic effects. This multi-model approach demonstrates the potential to uncover a comprehensive set of candidate genes associated with PHS.

### Z216 and Z33 exhibit differences in PHS phenotypes

**Figure 5:**
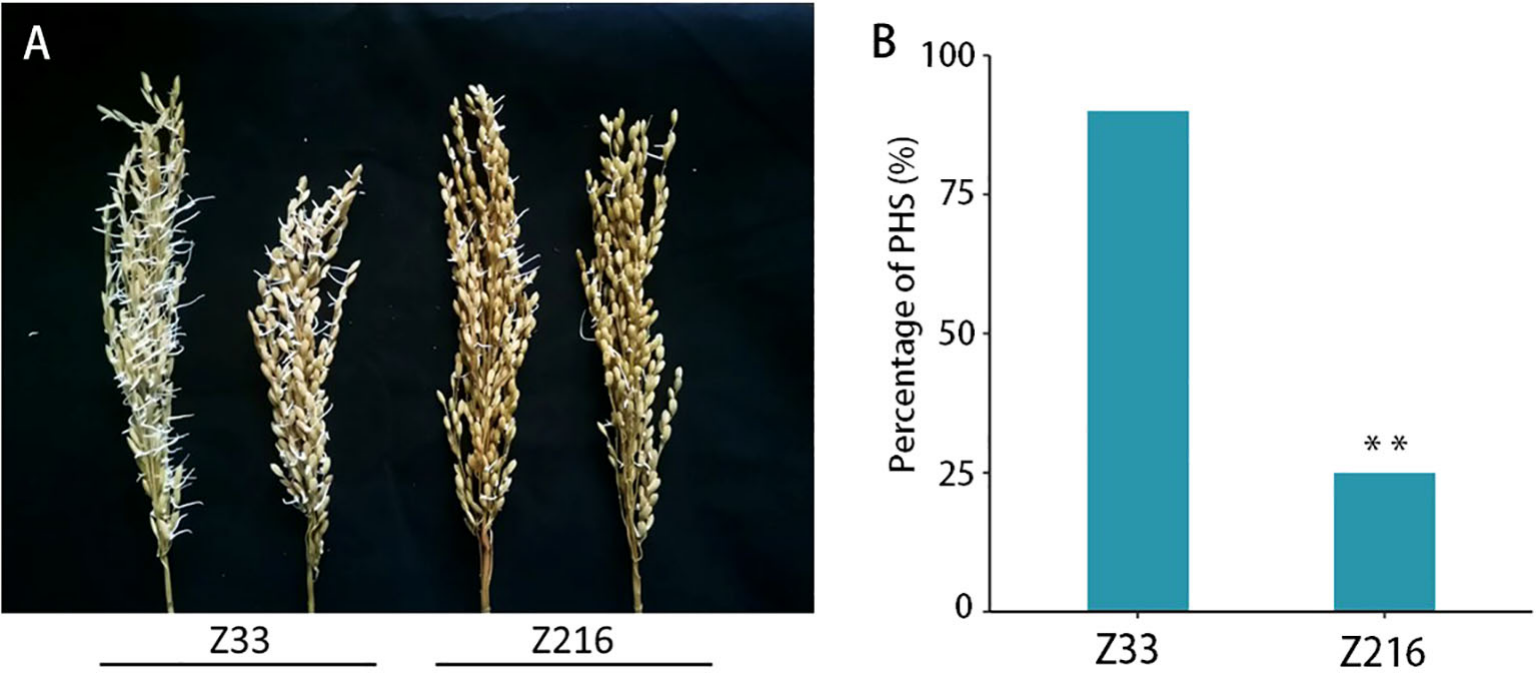
Comparison of the PHS phenotype in Z33 and Z216 cultivars under high humidity conditions. **(A)** PHS phenotype in Z33 and Z216 lines; **(B)** Comparison of PHS after 24 hours of high humidity treatment (n=5, T-test, **P < 0.01).

We further evaluated the PHS-resistance level of 165 rice accessions under high-humidity conditions and found significant differences in PHS levels between Z33 and Z216 cultivars (**Figure 5A**). At 24 hours, Z216 had an average germination rate of 24%, compared to Z33’s significantly higher rate of 90% (**Figure 5B**). This suggests that Z216 cultivar is more resistant to panicle sprouting, while Z33 cultivar is more sensitive.

### RNA-seq and DEGs analysis

**Figure 6:**
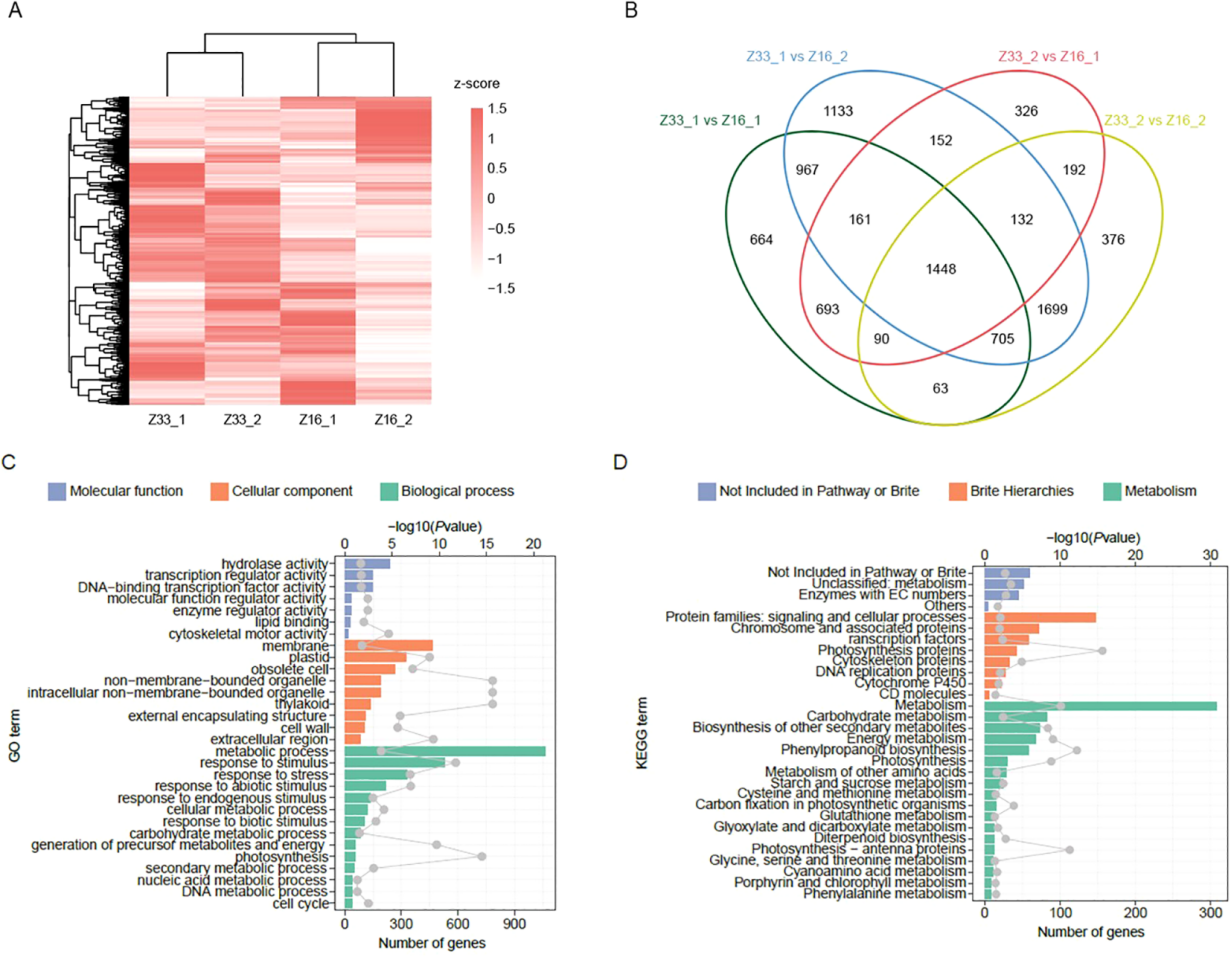
Transcriptome analysis of PHS between Z33 and Z216 cultivars. **(A)** Gene expression profile; **(B)** DEG analysis of Z33 and Z216 cultivars; **(C)** GO classification and **(D)** KEGG classification of Z33 and Z216.

To explore the expression of PHS regulated genes in rice, we conducted transcriptome sequencing on Z33 and Z216 cultivars following 24-hour high-humidity treatment. Through FPKM analysis, we detected a total of 25,776 expressed genes. Hierarchical clustering revealed distinct transcriptomic differences between the two materials (**Figure 6A**). A total of 19,087 DEGs were identified (**Figure 6B**), with Z33 having 3,975 upregulated and 7,213 down-regulated DEGs, and Z216 having 3,105 upregulated and 4,794 down-regulated DEGs. These DEGs overlapped with 21 previously reported PHS-related genes.

DEG analysis further highlighted the top three enriched GO terms as metabolic process (GO:0008152), stimulus response (GO:00508962), and membrane system (GO:0016020). The most enriched KEGG pathways were starch and sucrose metabolism (A09100), plant hormone signal transduction (B09183), and phenylpropanoid biosynthesis (B09101) (**Figures 6C, D**). The upregulated amylase genes (e.g., *LOC_Os08g36910*) accelerate endosperm breakdown to supply energy and carbon skeletons for germination, while differentially expressed ABA-related genes (e.g., *LOC_Os01g02120*, *LOC_Os01g64000*) can change the hormone balance. Analyzing the regulatory network offers new insights into PHS mechanisms, provides key targets for molecular breeding, and improves breeding efficiency for panicle sprouting resistance.

### Candidate gene identification and haplotype analysis

**Figure 7:**
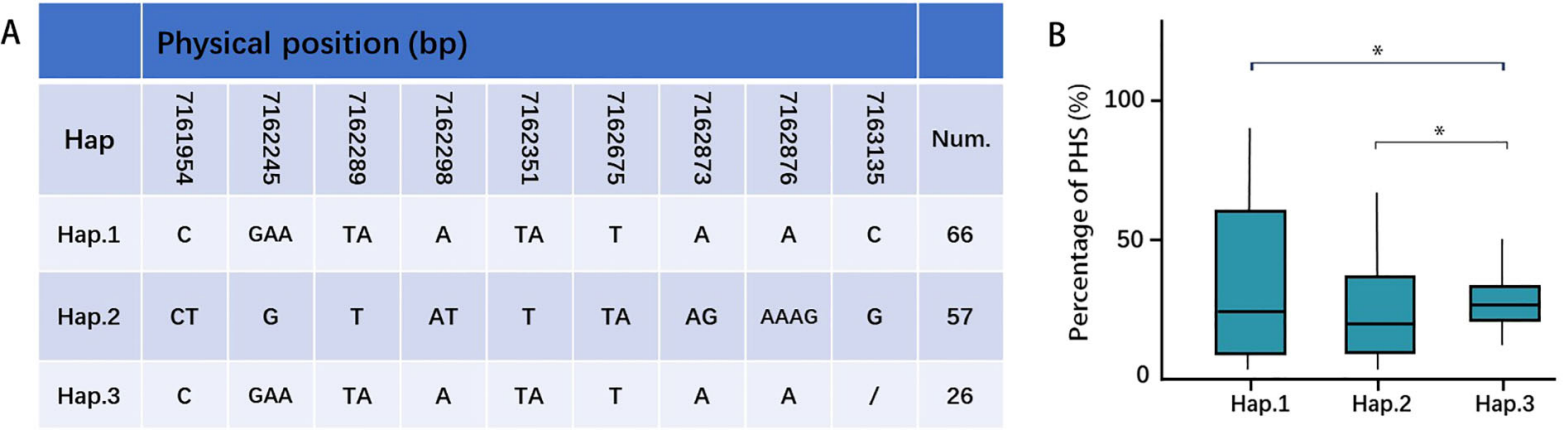
Haplotype analyses of *UGT74J1* gene. Gene structure **(A)** and PHS **(B)** of different haplotypes (T-test, *P < 0.05).

GWAS analysis has provided a new dataset of candidate genes for PHS-related, defining twenty-one candidate QTLs. These intervals contain several previously cloned PHS-related genes: *OsCDP3.10* (*LOC_Os03g57960*; cupin domain-containing protein; Huang et al., 2021), *OsWRKY50* (*LOC_Os11g02540*; transcription factor repressor; Peng et al., 2022), *UGT74J1* (*LOC_Os04g12980*; *UDP-glucosyltransferase*; Tezuka et al., 2021), *OsJAZ6* (*LOC_Os03g28940*; jasmonate ZIM-domain protein; Wang et al., 2024), and *IPA1* (*LOC_Os08g39890*; ideal plant architecture gene; Chen et al., 2023). Furthermore, integrating transcriptomic analysis facilitated further gene mining, revealing an overlap between differentially expressed genes (DEGs) and GWAS candidate genes. Specifically, *UGT74J1*, encoding a UDP-glucosyltransferase, was identified as a candidate gene on chromosome 4 through both MLM and MLMM models. Haplotype analysis, defined by a missense mutation in the coding region, identified *UGT74J1*-Hap3 as a superior haplotype conferring PHS resistance (**Figure 7**), highlighting the functional relevance of this coding sequence variation and underscoring the value of *UGT74J1* as a promising candidate for PHS-related marker development.

## Discussion

Pre-harvest sprouting, the premature germination of rice grains in in humid conditions before harvest, compromises grain quality and yield while elevating susceptibility to mold and pests, posing a threat to global food security (Lee et al., 2021; Sohn et al., 2021). Given that PHS susceptibility is influenced by both environmental factors and genetic background (Chen et al., 2021), identifying resistance-associated genetic variations across diverse germplasms offers significant potential for breeding improvement (He et al., 2021). This study therefore aimed to identify PHS-associated variants, candidate genes, and regulatory pathways to facilitate the development of resilient rice varieties.

We selected a population comprising *indica* accessions based on their high genetic diversity and agricultural relevance. *Indica* constitutes a predominant portion of worldwide rice output and is largely cultivated in the warm and humid environments of Asia, a climatic profile that strongly predisposes the crop to PHS. Nevertheless, the genetic foundations of PHS resistance in indica have been less thoroughly investigated relative to japonica. Therefore, the genetic enhancement of PHS resistance in indica emerges as a pressing breeding goal (Xu et al., 2025). We evaluated PHS susceptibility in a diverse panel of 165 *indica* rice accessions under controlled high-humidity conditions. Phenotypic assessment revealed a broad spectrum of PHS susceptibility, with cultivars Z33 and Z216 identified as highly susceptible and resistant extremes, respectively, and selected for further investigation. This careful phenotyping not only establishes a reliable foundation for subsequent genomic analyses but also identifies valuable germplasm donors, such as Z216 and Guang Xuan 3 Hao, which can be directly utilized in crossing programs to introduce PHS resistance into elite genetic backgrounds.

Whole-genome resequencing generated a high-density variation map of over 1.58 million high-quality SNPs. This dataset itself constitutes a valuable community resource, enabling future genetic studies and marker development in indica rice. GWAS using the MLMM, MLM, and FarmCPU models identified 21 significant loci associated with PHS. The co-detection of three loci by at least two independent models underscores the reliability of these associations. The integration of GWAS with transcriptomic data significantly refined our candidate gene list. This multi-omics strategy effectively shifted the focus from large genomic intervals to high-probability candidates, thereby enhancing the efficiency of gene discovery. Notably, several previously cloned PHS-related genes, including *OsCDP3.10* (Huang et al., 2021), *OsWRKY50* (Peng et al., 2022), *UGT74J1* (Tezuka et al., 2021), *OsJAZ6* (Wang et al., 2024), and *IPA1* (Chen et al., 2023), were located within our candidate intervals, which cross-validates the effectiveness of our approach. Among the novel candidates, *UGT74J1* emerged as a high-priority gene, being highlighted by both GWAS and differential expression analysis. The identification of *UGT74J1*-Hap3 as a superior haplotype associated with PHS resistance has direct and immediate implications for molecular breeding. This finding allows for the development of a co-dominant, functional marker based on the haplotype-specific SNPs. This marker can be used in MAS to rapidly introgress the resistant *UGT74J1*-Hap3 allele into susceptible elite varieties, significantly accelerating the breeding of PHS-resistant cultivars. This strategy is particularly crucial for *indica* rice improvement, as it enables the enhancement of a complex trait without compromising on other agronomic traits.

Transcriptomic profiling under PHS-inducing conditions revealed 19,087 DEGs in Z33 and Z216, revealing a complex regulatory network and highlighting significant enrichment in critical metabolic and stimulus-response pathways. Beyond confirming known pathways, our transcriptome data revealed specific regulatory nodes, such as the coordinated expression of amylase and ABA-related genes. This detailed view of the PHS-related regulatory network provides a theoretical foundation for pyramiding multiple favorable alleles across different pathways, which is a promising strategy for breeding durable and broad-spectrum PHS resistance.

In conclusion, this study elucidates the genetic architecture of pre-harvest sprouting resistance in indica rice through an integrated genomic, transcriptomic, and population genetic framework. Our findings provide actionable resources for breeding, including a high-density SNP dataset for genomic selection, superior haplotypes for marker-assisted selection, and characterized resistant germplasm. The deployment of these resources will accelerate the development of high-yielding, climate-resilient rice varieties, thereby contributing to global food security.

## Data Availability

The original contributions presented in the study are included in the article/**Supplementary Material**, further inquiries can be directed to the corresponding author/s. Genome data in this study have been deposited in the China National Genomics Data Center (https://ngdc.cncb.ac.cn/) under the project accession numbers PRJCA047790.
